# Polymorphisms of *FST* gene and their association with wool quality traits in Chinese Merino sheep

**DOI:** 10.1371/journal.pone.0174868

**Published:** 2017-04-06

**Authors:** Guang-Wei Ma, Yan-Kai Chu, Wen-Jian Zhang, Fei-Yue Qin, Song-Song Xu, Hua Yang, En-Guang Rong, Zhi-Qiang Du, Shou-Zhi Wang, Hui Li, Ning Wang

**Affiliations:** 1 Key Laboratory of Chicken Genetics and Breeding, Ministry of Agriculture, Harbin, P. R. China; 2 Key Laboratory of Animal Genetics, Breeding and Reproduction, Education Department of Heilongjiang Province, Harbin, P. R. China; 3 College of Animal Science and Technology, Northeast Agricultural University, Harbin, P. R. China; 4 Institute of Animal Husbandry and Veterinary Medicine, Xinjiang Academy of Agricultural and Reclamation Science, Shihezi, P. R. China; 5 State Key Laboratory of Agrobiotechnology, China Agricultural University, Beijing, P. R. China; University of Illinois, UNITED STATES

## Abstract

Follistatin (FST) is involved in hair follicle morphogenesis. However, its effects on hair traits are not clear. This study was designed to investigate the effects of *FST* gene single nucleotide polymorphisms (SNP) on wool quality traits in Chinese Merino sheep (Junken Type). We performed gene expression analysis, SNP detection, and association analysis of *FST* gene with sheep wool quality traits. The real-time RT-PCR analysis showed that *FST* gene was differentially expressed in adult skin between Chinese Merino sheep (Junken Type) and Suffolk sheep. Immunostaining showed that FST was localized in inner root sheath (IRS) and matrix of hair follicle (HF) in both SF and Suffolk sheep. Sequencing analysis identified a total of seven SNPs (termed SNPs 1–7) in the *FST* gene in Chinese Merino sheep (Junken Type). Association analysis showed that SNP2 (Chr 16. 25,633,662 G>A) was significantly associated with average wool fiber diameter, wool fineness SD, and wool crimp (*P* < 0.05). SNP4 (Chr 16. 25,633,569 C>T) was significantly associated with wool fineness SD and CV of fiber diameter (*P* < 0.05). Similarly, the haplotypes derived from these seven identified SNPs were also significantly associated with average wool fiber diameter, wool fineness SD, CV of fiber diameter, and wool crimp (*P* < 0.05). Our results suggest that FST influences wool quality traits and its SNPs 2 and 4 might be useful markers for marker-assisted selection and sheep breeding.

## Introduction

Sheep (*Ovis aries*) play an important role in the global agriculture economy being one of the earliest domesticated animal. Sheep are raised for wool, meat, skin and milk. A number of sheep breeds have been bred for different purposes. The Merino is an old and influential sheep breed, and Merino wool is the finest and softest wool obtained from sheep. Chinese Merino sheep (Junken Type) breeding started in 1972 and, to date, six strains of Chinese Merino sheep (Junken Type) have been developed for various purposes: the superfine wool strain (SF), the prolific wool strain (PW), the prolific meat strain (PM), and strains A, B, and U [1]. Strain A was selected for large body size and high wool yield, strain B was selected for long wool fiber, and strain U was selected for long wool fiber, high wool yield, and lower fiber diameter [1].

Wool quality traits are polygenic traits, and several of genes have been reported to be associated with sheep wool quality traits. Two SNPs in keratin-associated protein 8.1 (*KAP 8*.*1*) gene and one SNP in keratin-associated protein 1.3 (*KAP 1*.*3*) gene were shown to be associated with average wool fiber diameter in Chinese Merino sheep [2]. A 57-bp deletion of keratin-associated protein 6.1 (*KAP 6*.*1*) gene was associated with wool fineness SD and CV of fiber diameter in Southdown × Merino cross lambs [3]. Two SNPs in keratin-associated protein 22.1 (*KAP 22*.*1*) gene and six SNPs in desmoglein 4 (*DSG 4*) gene were shown to influence wool crimp in Southdown × Merino cross lambs and Tan sheep, respectively [4, 5]. In addition, our previous study showed that nine SNPs of methionine synthase (*MTR*) gene were associated with average wool fiber diameter in Chinese Merino sheep [6].

Follistatin (FST) was first isolated from bovine and porcine ovarian follicular fluid in 1987 [7, 8]. *FST* gene is located on human chromosome 5, mouse chromosome 13, cow chromosome 20, and sheep chromosome 16. Ovine *FST* gene is a relatively small gene consisting of six exons spanning around 6 kb of genomic region and encodes two protein isoforms due to the alternative splicing: FST344 and FST317. Signal peptide removal of FST344 and FST317 yields mature polypeptides of 315 (FST315) and 288 (FST288), respectively. As a secreted glycoprotein, FST inhibits activin activity by two mechanisms. First, FST288 binds to activin at the membrane surface to form the follistatin/activin complex, and then this complex is endocytosed and degraded by the lysosome. Second, FST315 binds to activin and prevents activin binding to its receptors [9, 10].

*FST* is widely expressed in mammalian tissues and organs, such as ovary, skin, and skeletal muscle [9, 11, 12]. *In situ* hybridization analysis showed that *FST* gene transcript was expressed in hair placode, outer root sheath, and interfollicular epidermis in mouse embryonic hair follicles [13]. Further immunostaining showed that the FST was expressed in the hair placode, matrix, and inner root sheath as well as the interfollicular epidermis in mouse embryonic hair follicles [13]. It has been shown that FST is involved in the regulation of hair follicle morphogenesis. Knockout mouse studies showed that homozygous *FST* knockout mice died shortly after birth because of impaired lung development [14]. Histological analysis showed that, at 18.5 days post coitus, *FST* knockout embryos had a higher percentage of hair follicles in the early stages (stages 1 and 2) and a lower percentage of hair follicles in more advanced stages (stages 3 and 4) in the back skin, compared with their littermate control embryos [13], indicating that *FST* knockout retards mouse hair follicle morphogenesis. Compared with their littermate control, the homozygous *FST* knockout newborn mice had curlier whiskers [14]. A transgenic mouse study showed that compared with their littermate control, *FST* transgenic mice had normal body weight and body size, but shinier and more irregular hair [15]. Furthermore, an epidermis-specific transgenic overexpression of *FST* caused thinner epidermis, smaller hair follicles, and more irregular hair [16].

Although it has been demonstrated that FST regulates hair follicle morphogenesis [13], its effects on hair traits are not clear. Here, to study the effects of *FST* gene on hair traits, we performed gene expression analysis as well as single nucleotide polymorphisms detection followed by association analysis of *FST* gene with sheep wool quality traits in Chinese Merino sheep (Junken Type).

## Materials and methods

### Ethics statement

All animal work was conducted according to the guidelines for the care and use of experimental animals established by the Ministry of Science and Technology of the People’s Republic of China (Approval number: 2006–398), and was approved by the Laboratory Animal Management Committee of Northeast Agricultural University.

### Animal resource population and phenotyping

A total of 744 Chinese Merino sheep (Junken Type) were used in this study, from the Xinjiang Academy of Agricultural and Reclamation Science. These sheep were from six strains of Chinese Merino sheep (Junken Type) (strain **A** (n = 152), strain **B** (n = 103), the prolific meat strain (**PM**, n = 134), the prolific wool strain (**PW**, n = 138), the superfine wool strain (**SF**, n = 181) and strain **U** (n = 36)). Strain A was selected for large body size and high wool yield, strain B was selected for long wool fiber, and strain U was selected for long wool fiber, high wool yield, and lower fiber diameter [1]. In addition, sixteen ewes (eight from each of SF and Suffolk sheep) were used for *FST* gene expression analysis. Of these sixteen sheep, the three SF strain sheep were slaughtered at 240 d of age, and the body side skin, skeletal muscle, small intestine, ovary, heart, liver, spleen, pituitary gland, kidney, rumen, and pineal gland samples were collected and other five SF strain sheep were slaughtered at 240 d of age for collecting only body side skin samples. The eight Suffolk sheep were slaughtered at 240 d of age for collecting only body side skin samples. All collected tissue samples were snap-frozen in liquid nitrogen and stored at—80°C until analyzed. All sheep were kept in the same environmental conditions and had free access to feed and water. The ear notch samples and wool samples were collected at shearing. The wool quality traits were measured following the guidelines of the China Fiber Inspection Bureau and International Wool Textile Organization [17]. A total of five traits were measured and recorded: average wool fiber diameter, wool fineness SD, CV of fiber diameter, wool crimp, and wool fiber length.

### DNA and RNA extraction and cDNA synthesis

Genomic DNA from the 744 samples was isolated from ear tissue samples using the phenol—chloroform method and stored at—20°C for genotyping [18]. Total RNA from the frozen tissues was isolated with Trizol reagent (Invitrogen, Rockville, MD) according to the instructions, and RNA quality was assessed by denaturing formaldehyde agarose gel electrophoresis. Total RNA (1μg) was reverse transcribed to cDNA using Promega Improm-II reverse transcription System (Promega, Madison, WI) following the instructions.

### Real-time reverse transcription-PCR

Real-time Reverse Transcription-PCR (real-time RT-PCR) was performed on an Applied Biosystems 7500 Fast Real-Time PCR Systems (US) using the Taq SYBR^®^ Green qPCR Premix (NOVA, Yugong Biolabs Inc., Lianyungang, Jiangsu, China). Thermal cycling consisted of an initial step at 95°C for 10 min followed by 40 cycles at 95°C for 30 s and 60°C for 30 s. The following primers were used: *FST* gene, forward primer: 5'-GCCGAATGAACAAGAAGAACAAAC-3' and reverse primer: 5'-TCAGGTGACAGGCACTGGGGTA-3' and *GAPDH* gene, forward primer: 5'-CTGACCTGCCGCCTGGAGAAA-3' and reverse primer: 5'-GTAGAAGAGTGAGTGTCGCTGTT-3'. *FST* gene expression was quantified relative to *GAPDH* gene expression by the 2^-ΔCt^, where ΔCt = Ct_FST_−Ct_GAPDH_. *GAPDH* gene was served as internal standard for normalization. Data were analyzed with SAS 8.0, in which a *t*-test or ANOVA procedure to examine the significance of difference in gene expression. *P*-value < 0.05 was considered as statistically significant and *P*-value < 0.01 was considered as highly significant, unless otherwise specified.

### Histology and immunostaining

The body side skin was collected from six ewes (three from each of SF and Suffolk sheep). Skin samples were fixed in 10% neutral-buffered formalin, embedded in paraffin, sectioned, and stained with hematoxylin and eosin. Paraffin embedded sections were stained following a standard immunohistochemical staining protocol. Paraffin sections (8 μm thick) were blocked with 10% goat serum (room temperature, 20 min), and were incubated overnight at 4°C with polyclonal rabbit anti-human FST antibody (sc-30194, Lot #A1644. Santa Cruz Biotechnology, Inc.) at a 1:100 dilution. After rinsed in phosphate buffer saline, paraffin sections were incubated with biotin conjugated goat anti-rabbit IgG secondary antibody (BOSTER, China) at a 1:1000 dilution for 1 h at 37°C. Labeling was visualized by DAB kit (BOSTER, China). The images were taken under a Nikon microscope (ECLIPSE 80i, Japan).

### Single nucleotide polymorphism detection in the ovine *FST* gene

In an effort to identify SNP in a cost-effective manner, SNP discovery was achieved by sequencing PCR products of the pooled DNA samples from 60 Chinese Merino sheep (Junken Type) individuals. Two partial regions (1280 bp and 2101 bp) of the *FST* gene were amplified by PCR using primer pairs FST-1 and FST-2, respectively (Table 1). The PCR products were purified and sequenced by Invitrogen (Shanghai, China). The sequences were aligned using the Align X function of Vector NTI (Informax, Rockville, MD). SNPs were identified by double peaks at a single base in the chromatograms.

**Table 1:** Primers used for amplification of the ovine *FST* gene.

Primer pair	Primer sequence (5'- 3')	*FST* gene regions	Amplified genomic position	Product size
FST-1	F: TCAGGTCCTGTACAAGACAGAACTG	Exons 2–4 and Introns 2 and 3	Chr16: 25,632,658–25,633,937	1280 bp
	R: ACTGGGGTAGGTCACTCCATCATT			
FST-2	F: GACACTAAAGGTTCAACCTAGGGTC	Introns 3–5 and Exons 4–6	Chr16: 25,630,892–25,632,992	2101 bp
	R: TGTAGTCCTGGTCTTCATCTTCCTC			

### Genotyping of the ovine *FST* gene

A multiplexed SNP single base extension (SBE) assay was designed by using Sequenom Assay Design 3.1 software (Sequenom, San Diego, CA) according to the instructions. Genotyping was performed by using a 384-well plate format on the Sequenom MassARRAY platform (Bioyong Technologies Inc., Beijing, China) during the year 2012. The raw data files generated by Mass Array Sequenom were analyzed for the intensity peaks of calibrant to ascertain the quality of the data as previous described [19]. An overall call rate of >95% was maintained. For every 96 samples (a quadrant of the Sequenom chip), four samples were duplicated and the call rates were checked for concordance. The calls in the negative control (no DNA) were also monitored in all the runs. Reproducibility was 100% in the present study.

### Statistical analysis

Correlation analysis between *FST* gene expression in skin and average wool fiber diameter was subjected to the Pearson procedure of IBM SPSS 19.0 (US). Genotype and allele frequencies were subjected to the Descriptive statistics of IBM SPSS 19.0 (US). The Hardy-Weinberg equilibrium using χ ^2^ test of each SNP was performed by IBM SPSS 19.0 (US). The expected heterozygosity and Shannon's information index (*I*) were performed by the Popgene 32 software (Version 1.31). The polymorphism information content (*PIC*) and observed heterozygosity were calculated according to the allele process of the SAS 9.1.3 genetics software package. The graphical representation of the Linkage disequilibrium (LD) structure was performed by the Haploview software (version 4.2). The haplotype analysis was conducted in SAS/GENETICS using the PROC HAPLOTYPE procedure with a sliding window (S1 Table). This procedure uses the Expectation Maximization (EM) algorithm to generate maximum likelihood estimates of the haplotype frequencies.

Before analyzing the association between the identified SNPs and wool quality traits, we performed the data preprocessing: if the number of one genotype was fewer than 5% × the total number of samples, we removed the data for this genotype.

Based on the characteristics of Chinese Merino sheep (Junken Type), the statistical models were:

Model 1:Y = μ + G + L + A + G × L + G × A + A × L + e,

Model 2: Y = μ + H + L + A + H × L + H × A + A × L + e,

where *Y* is the phenotype value; *μ* is the population mean; genotype (*G*), haplotype (*H*), line (*L*), and age (*A*) were the fixed effects; *G × L*, *G × A*, and *A × L* were the interaction effect of *G* by *L*, *G* by *A*, and *A* by *L*; *H × L*, *H × A*, and *A × L* were the interaction effect of *H* by *L*, *H* by *A*, and *A* by *L*; and *e* was the residual effect. Data were subjected to the GLM procedures of John’s Macintosh Program 7.0 (JMP, SAS Inst. Inc., Cary, NC), which was used to examine the correlation between genotypes and haplotypes and continuous traits and to evaluate the least squares means. *P*-value < 0.05 was considered statistically significant, and *P*-value < 0.01 was highly significant, unless otherwise specified.

## Results

### Ovine *FST* gene expression

**Fig 1:**
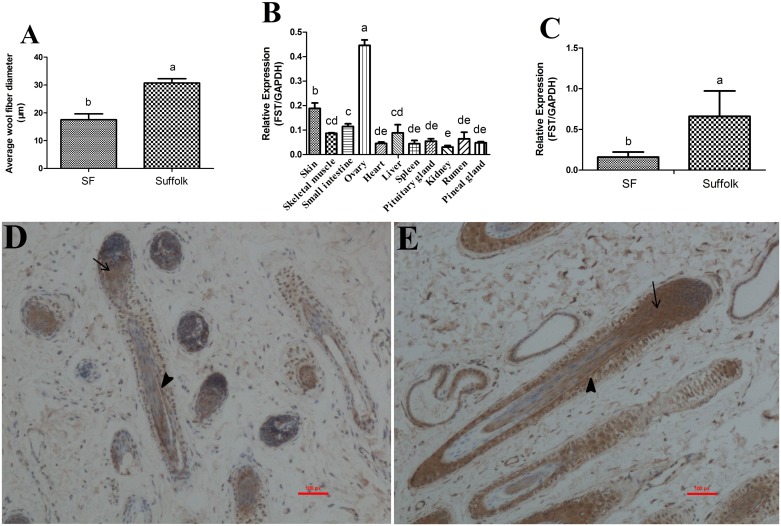
Ovine *FST* gene expression. (**A**) The average wool fiber diameter of SF (n = 3) and Suffolk (n = 3) sheep. In a row with different superscripts differ significantly (*P* < 0.05). (**B**) The *FST* gene expression in various tissues of the SF sheep (n = 3). The housekeeping gene *GAPDH* was used as an internal control for real-time RT-PCR analysis. In a row with different superscripts differ significantly (*P* < 0.05). (**C**) The *FST* gene expression levels in the body side skin between SF (n = 8) and Suffolk (n = 8). The housekeeping gene *GAPDH* was used as an internal control for real-time RT-PCR analysis. In a row with different superscripts differ significantly (*P* < 0.05). (**D**) Immunohistochemical localization of FST in SF sheep skin (n = 3). (**E**) Immunohistochemical localization of FST in Suffolk sheep skin (n = 3). The inner root sheath, arrowhead; matrix, arrow.

Merino and Suffolk are two different sheep breeds. There is a dramatic difference in average wool fiber diameter between the two sheep breeds [20]. Consistent with the known wool quality difference between Merino and Suffolk, our wool fiber measurement showed that the superfine wool strain (**SF**) Merino had lower average wool fiber diameter than Suffolk sheep (SF sheep: 17.58 μm; Suffolk: 30.92 μm; *P* = 0.0009; Fig 1A). To obtain the clue for understanding the effects of *FST* gene on sheep wool quality traits, we performed the tissue expression analysis of *FST* gene in SF sheep and Suffolk sheep by real-time RT-PCR. The results showed that *FST* gene was ubiquitously expressed in the eleven tested adult tissues of SF sheep (Fig 1B), and comparatively highly expressed in the body side skin and ovary (Fig 1B). Skin expression comparison revealed that *FST* gene expression was 4.15-fold lower in SF sheep than in Suffolk sheep (*P* < 0.05; Fig 1C). Correlation analysis showed that skin *FST* gene expression was positively significantly correlated to the average wool fiber diameter (Pearson’s *r* = 0.987, *P <* 0.05), suggesting that *FST* gene expression variation may contribute to the variation of wool quality traits between SF sheep and Suffolk sheep. Immunohistochemical localization showed that FST was localized in inner root sheath (IRS) and matrix of hair follicle (HF) in both SF and Suffolk sheep (Fig 1D and 1E).

### Identification of SNPs

Sequencing analysis identified a total of seven SNPs (termed SNPs 1–7) in the two overlapping fragments of the ovine *FST* gene in Chinese Merino sheep (Junken Type). Of these seven SNPs, four (SNPs 1–4) were located in intron 2 of the *FST* gene, one (SNP5, S216T) was located in exon 4, and two (SNPs 6 and 7) were located in intron 4. The nomenclature and distribution of the seven identified SNPs were presented in Table 2.

**Table 2:** Summary of the seven identified SNPs in ovine *FST* gene.

SNP ID ^1^	Chromosome position ^2^, bp	Locations	db SNP rs # ID ^3^	AA exchange
SNP1	Chr 16. 25,633,722	Intron 2	rs403714633	
SNP2	Chr 16. 25,633,662	Intron 2	rs425207127	
SNP3	Chr 16. 25,633,632	Intron 2	rs401759231	
SNP4	Chr 16. 25,633,569	Intron 2	rs423028521	
SNP5	Chr 16. 25,632,659	Exon 4	rs161143581	S216T
SNP6	Chr 16. 25,632,504	Intron 4	rs598552142	
SNP7	Chr 16. 25,632,294	Intron 4	rs403563544	

^1^ ID = identifier.

^2^ Nucleotides are numbered according to the *Ovis aries* genome ([ISGC Oar_v3.1/oviAri3], http://genome.ucsc.edu/cgi-bin/hgBlat) and located on chromosome 16 at 25,630,892 to 25,633,937 bp, bp = base pair.

^3^ db = database; rs# = reference SNP #.

### Frequency of alleles and genotypes

We genotyped these seven identified SNPs in 744 individuals of six Chinese Merino sheep (Junken Type) strains (A, B, PM, PW, SF and U) using the SBE assay. In the tested population, three genotypes were found for each of these seven identified SNPs (S2 Table). The MAF of these seven SNPs ranged from 4.76% to 41.83% (Table 3). The observed heterozygosities and expected heterozygosity of these seven SNPs ranged from 0.0237 to 0.4533 and 0.0907 to 0.4867, respectively. Except SNP6, the observed heterozygosities of all other SNPs were lower than the expected heterozygosity (Table 3). The allele frequencies observed for the seven identified SNPs in the six tested strains were summarized in S2 Table. The polymorphism information content (*PIC*) analysis showed that at species level, SNPs 1–3, SNPs 5 and 6 were in low diversity (*PIC* < 0.25), and SNPs 4 and 7 were in moderate diversity (0.25 < *PIC* < 0.5) (Table 4), but at population level, except SNP7 which was in moderate diversity (0.25 < *PIC* < 0.5), the other six SNPs were almost in low diversity (*PIC* < 0.25) (Table 4). We also performed the Shannon's information index (*I*) test for the seven identified SNPs in the six tested strains (Table 5). The results showed that, compared to other six SNPs, SNP7 had the largest *I* score at species and population levels, suggesting that the tested population had high genetic diversity based on SNP7 data (Table 5). Statistical analysis showed that the *PIC* was positively significantly correlated with the *I* score (Pearson’ s r = 0.999, *P* < 0.05). The χ ^2^ test showed that among these seven identified SNPs, SNPs 2 and 3 were not in Hardy-Weinberg equilibrium based on the combined data (Table 3, *P* < 0.05).

**Table 3:** Summarized information for the seven identified SNPs in ovine *FST* gene.

SNP ID	Alleles	MAF ^1^	Het.Obs. ^2^	Het.Exp. ^3^	HW ^4^ *P*-value
SNP1	*G* / *A*	0.0628	0.1093	0.1178	0.0510
SNP2	*G / A*	0.1950	0.0237	0.3139	2.089×10−^155^
SNP3	*A / C*	0.0476	0.084	0.0907	0.0494
SNP4	*C / T*	0.1881	0.2969	0.3054	0.4475
SNP5	*C / G*	0.0605	0.1073	0.1137	0.1274
SNP6	*G / A*	0.0527	0.1027	0.0999	0.4371
SNP7	*C / T*	0.4183	0.4533	0.4867	0.0935

^1^ MAF = minor allele frequency. A/B implies that B is the minor allele.

^2^ Het.Obs. = Observed heterozygosity.

^3^ Het.Exp. = Expected heterozygosity.

^4^ HW = Hardy-Weinberg equilibrium.

**Table 4:** Polymorphism Information Content (*PIC*) for the seven identified SNPs in Chinese Merino sheep (Junken Type) population.

Strain	SNP1	SNP2	SNP3	SNP4	SNP5	SNP6	SNP7
A	0.0382	0.1751	0.0263	0.0733	0.0390	0.0733	0.3501
B	0.1435	0.2915	0.1291	0.1860	0.1491	0.0097	0.3386
PM	0.2047	0.3188	0.1392	0.1491	0.1891	0.0434	0.3398
PW	0.0923	0.1837	0.0813	0.3216	0.0929	0.2653	0.3744
SF	0.0869	0.2983	0.0742	0.3562	0.0826	0.0270	0.3704
U	0.0787	0.3047	0.0787	0.2150	0.0767	0.0000	0.3627
Species level	0.1108	0.2469	0.0866	0.2588	0.1072	0.0949	0.3682
Population level	0.1074	0.2620	0.0881	0.2169	0.1049	0.0698	0.3560

Polymorphism information content = *PIC*. *PIC* < 0.25 means low diversity; 0.25 < *PIC* < 0.5 means moderate diversity; *PIC* > 0.5 means high diversity.

**Table 5:** Shannon's information Index (*I*) content for the seven identified SNPs in Chinese Merino sheep (Junken Type) population.

Strain	SNP1	SNP2	SNP3	SNP4	SNP5	SNP6	SNP7
A	0.0975	0.3441	0.0716	0.1671	0.0991	0.1671	0.6448
B	0.2908	0.5393	0.2664	0.3622	0.3005	0.0307	0.6234
PM	0.3932	0.5874	0.2835	0.3006	0.3672	0.1085	0.6255
PW	0.2017	0.3582	0.1818	0.5924	0.2028	0.4943	0.6920
U	0.1769	0.5623	0.1769	0.4101	0.1732	0.0000	0.6690
SF	0.1918	0.5512	0.1687	0.6563	0.1841	0.0732	0.6840
Species level	0.2347	0.4934	0.1914	0.4835	0.2284	0.2064	0.6797
Population level	0.2253	0.4904	0.1915	0.4148	0.2212	0.1456	0.6565

### Association of SNPs in the *FST* gene with wool quality traits

Using JMP 7.0, we analyzed the association between the identified SNPs and wool quality traits using the linear model 1. As shown in Table 6, in the tested Chinese Merino sheep (Junken Type) population tested, SNP2 was significantly associated with the average wool fiber diameter and wool crimp, and SNP4 was significantly associated with wool fineness SD (*P* < 0.05). In addition, SNP2 was highly significantly associated with wool fineness SD, and SNP4 was highly significantly associated with and CV of fiber diameter (Table 6, *P* < 0.01). Sheep with the *GG* genotype of SNP2 had significantly higher average wool fiber diameter and wool fineness SD but significantly lower wool crimp than the sheep with *AA* genotype (Table 6, *P* < 0.05). Sheep with the *CC* genotype of SNP4 had significantly lower wool fineness SD and CV of fiber diameter than the sheep with *CT* genotype (Table 6, *P* < 0.05).

**Table 6:** Effects (least squares means) of *FST* gene genotypes on wool quality traits. Only traits associated with the identified SNPs were presented in this table.

SNP ID ^1^	Traits	*P*-value	Genotype (number)	
			GG (577)	AA (139)
SNP2	Average wool fiber diameter, μm	0.0171 *	20.6885 (0.0856) ^a^	20.2247 (0.1759) ^b^
	Wool fineness SD, μm	0.0035 **	4.1803 (0.0295) ^a^	3.9847 (0.0606) ^b^
	Wool crimp, crimps / 2.5 cm	0.0144 *	12.1063 (0.0966) ^b^	12.6436 (0.1985) ^a^
			CC (485)	CT (217)
SNP4	Wool fineness SD, μm	0.0212 *	4.1235 (0.0312) ^b^	4.2738 (0.0580) ^a^
	CV of fiber diameter, %	0.0078 **	19.9349 (0.1387) ^b^	20.7057 (0.2575) ^a^

^a, b^ Means in a row with different superscripts differ significantly (*P* < 0.05).

^1^ ID = identifier.

* *P* < 0.05;

** *P* < 0.01.

### Association of *FST* gene haplotypes with wool quality traits

The LD analysis showed that the seven identified SNPs were very close but not in complete LD (S1 Fig), so the haplotype analysis was conducted in SAS/GENETICS with a sliding window (S1 Table). The haplotype frequencies were showed in S3 Table. Using JMP 7.0, we analyzed the association between *FST* gene haplotypes and wool quality traits using the linear model 2. The effects (*P*—value) of the *FST* gene haplotypes on wool quality traits in the tested Chinese Merino sheep population were shown in Table 7. Haplotype 1 (SNP1 and SNP2) and Haplotype 2 (SNP2 and SNP3) were highly significantly associated with average wool fiber diameter, wool fineness SD, and wool crimp (Table 7, *P* < 0.01). Haplotype 3 (SNP3 and SNP4) and Haplotype 4 (SNP4 and SNP5) were significantly associated with wool fineness SD and CV of fiber diameter (Table 7, *P* < 0.05).

**Table 7:** Effects (least squares means) of *FST* gene haplotypes on wool quality traits. Only traits associated with the identified haplotypes were presented in this table.

Haplotype	Average wool fiber diameter, μm	Wool fineness SD, μm	CV of fiber diameter, %	Wool crimp, crimps / 2.5 cm
Haplotype 1	0.0044**	0.0012**		0.0038**
Haplotype 2	0.0019**	0.0005**		0.0038**
Haplotype 3		0.0227*	0.023*	
Haplotype 4		0.0331*	0.0248*	

* *P* < 0.05;

** *P* < 0.01.

## Discussion

Merino and Suffolk have striking difference in wool quality traits. Merino sheep produce the finest and softest wool, while the Suffolk sheep produce coarse wool. Here, we observed that *FST* gene was differentially expressed in the skin between Chinese Merino sheep (SF strain) and Suffolk sheep. SNP and haplotype association analyses showed that *FST* gene polymorphisms were associated with average wool fiber diameter, wool fineness SD, CV of fiber diameter and wool crimp in Chinese Merino sheep (Junken Type).

In the present study, our association analysis showed that *FST* gene was associated with several sheep wool quality traits. FST knockout and transgenic mouse studies have shown FST regulates hair follicle morphogenesis and cycling [13–16]. Hair or wool is the product of hair follicle which determines hair phenotype [10]. Therefore, we presumed that FST affect hair quality phenotype by regulating hair follicle morphogenesis and cycling. FST functions by inhibiting activin activity [10]. As expected, knockout and transgenic mouse studies showed that activin A also influences hair follicle morphogensis and hair phenotype [21, 22, 13]. Considering all the above mentioned observations, it was reasonable to conclude that *FST* gene may be a major gene affecting wool quality traits.

There is a negative correlation between average wool fiber diameter and wool crimp in sheep [23]. In our tested sheep population, SNP2 was significantly associated with both wool fiber diameter and wool crimp. Sheep with the *GG* genotype of SNP2 had significantly higher average wool fiber diameter and wool fineness SD but significantly lower wool crimp than the sheep with *AA* genotype (Table 6, *P* < 0.05). These data suggested that SNP2 could be a molecular marker for marker-assisted selection (MAS) for high quality wool in sheep. However, this needs validation in large sheep populations.

We observed that SNPs 2 and 3 were not in Hardy-Weinberg equilibrium (Table 3) in the tested Merino population. This phenomenon may be explained by the three possible reasons. First, the number of specific genotypes (AG of SNP2 and CC of SNP3) was too small probable due to genetic drift [24]. Second, the economically favorable traits (such as low average wool fiber diameter) have been artificially selected and SNPs 2 and 3 as the beneficial SNPs or in linkage equilibrium with the beneficial SNPs might have been accumulated [25]. Last, the individual with different genotype may have different environmental adaptability and the individual with low environmental adaptability may be eliminated by natural selection [26].

We previously performed GWAS on the same Chinese Merino sheep (Junken Type) population using the Illumina sheep SNP50 BeadChip [27], but our GWAS results did not detect the association of *FST* gene and wool quality traits. This discrepancy may be due to the low density of Illumina sheep SNP50 BeadChip, the high false negative rate of GWAS [28] or different statistical algorithm [27].

Of these seven identified SNPs, only SNPs 2 and 4 were associated with wool quality traits. These two intronic SNPs may be functional SNPs, or in close linkage with causative alleles that affect *FST* gene expression, resulting in altered wool quality traits. It has been reported that functional intronic SNPs can affect gene expression by several mechanisms. For example, some of these SNPs affect the transcriptional activity by altering transcription factor binding sites and some of them affect mRNA splicing efficiency [29, 30], or instead to alter the expression of alternative transcripts [31, 32]. It is worth investigating whether and how SNPs 2 and 4 affect *FST* gene expression and lead to variations in sheep wool quality traits in the future.

## Conclusions

In this study, a total of seven SNPs were identified in the ovine *FST* gene, and association analysis showed that SNP2 and SNP4 were associated with average wool fiber diameter, wool fineness SD, CV of fiber diameter and wool crimp. SNPs 2 and 4 may be useful for marker-assisted selection for high quality wool in Chinese Merino sheep (Junken Type).

## Supporting information

S1 Table The sliding windows for haplotype construction. (DOCX)

S2 Table Allele and genotype frequencies of the identified SNPs in Chinese Merino sheep (Junken Type). (DOCX)

S3 Table Haplotype frequencies of the identified SNPs in Chinese Merino sheep (Junken Type). (DOCX)

S1 Fig LD analysis of the seven identified SNPs in *FST* gene. (TIF)
