# Supplementary material for: Polymorphisms of FST gene and their association with wool quality traits in Chinese Merino sheep
Source: PLoS One. 2017 Apr 6;12(4):e0174868. doi: 10.1371/journal.pone.0174868 (PMC5383234; doi:10.1371/journal.pone.0174868)
Supplement: S1 Table — (DOCX) [file pone.0174868.s001.docx]

**S1 Table. The sliding windows for haplotype construction**

| Haplotype | SNPs |
| --- | --- |
| Haplotype 1 | SNP 1 and SNP 2 |
| Haplotype 2 | SNP 2 and SNP 3 |
| Haplotype 3 | SNP 3 and SNP 4 |
| Haplotype 4 | SNP 4 and SNP 5 |
| Haplotype 5 | SNP 5 and SNP 6 |
| Haplotype 6 | SNP 6 and SNP 7 |
