# Supplementary material for: Polymorphisms of FST gene and their association with wool quality traits in Chinese Merino sheep
Source: PLoS One. 2017 Apr 6;12(4):e0174868. doi: 10.1371/journal.pone.0174868 (PMC5383234; doi:10.1371/journal.pone.0174868)
Supplement: S2 Table — (DOCX) [file pone.0174868.s002.docx]

**S2 Table. Allele and genotype frequencies of the identified SNPs in Chinese Merino sheep (Junken Type)**

| SNP | Strain | Sample number | Genotype frequency | Number |  |  |  |  | Allele frequency |  |
| --- | --- | --- | --- | --- | --- | --- | --- | --- | --- | --- |
|  |  |  | GG |  | AG |  | AA |  | G | A |
| SNP 1 | A | 151 | 0.9603 | 145 | 0.0397 | 6 | 0.0000 | 0 | 0.9801 | 0.0199 |
|  | B | 100 | 0.8400 | 84 | 0.1500 | 15 | 0.0100 | 1 | 0.9150 | 0.0850 |
|  | PM | 131 | 0.7557 | 99 | 0.2214 | 29 | 0.0229 | 3 | 0.8664 | 0.1336 |
|  | PW | 137 | 0.9051 | 124 | 0.0876 | 12 | 0.0073 | 1 | 0.9489 | 0.0511 |
|  | SF | 178 | 0.9101 | 162 | 0.0843 | 15 | 0.0056 | 1 | 0.9522 | 0.0478 |
|  | U | 35 | 0.9143 | 32 | 0.0857 | 3 | 0.0000 | 0 | 0.9571 | 0.0429 |
|  | Total | 732 | 0.8825 | 646 | 0.1093 | 80 | 0.0082 | 6 | 0.9372 | 0.0628 |
|  |  |  | GG |  | AG |  | AA |  | G | A |
| SNP 2 | A | 147 | 0.8912 | 131 | 0.0000 | 0 | 0.1088 | 16 | 0.8912 | 0.1088 |
|  | B | 100 | 0.7700 | 77 | 0.0000 | 0 | 0.2300 | 23 | 0.7700 | 0.2300 |
|  | PM | 124 | 0.7258 | 90 | 0.0000 | 0 | 0.2742 | 34 | 0.7258 | 0.2742 |
|  | PW | 134 | 0.8806 | 118 | 0.0075 | 1 | 0.1119 | 15 | 0.8843 | 0.1157 |
|  | SF | 177 | 0.7571 | 134 | 0.0056 | 1 | 0.2373 | 42 | 0.7599 | 0.2401 |
|  | U | 36 | 0.7500 | 27 | 0.0000 | 0 | 0.2500 | 9 | 0.7500 | 0.2500 |
|  | Total | 718 | 0.8036 | 577 | 0.0028 | 2 | 0.1936 | 139 | 0.8050 | 0.1950 |
|  |  |  | AA |  | AC |  | CC |  | A | C |
| SNP 3 | A | 148 | 0.9730 | 144 | 0.0270 | 4 | 0.0000 | 0 | 0.9865 | 0.0135 |
|  | B | 100 | 0.8600 | 86 | 0.1300 | 13 | 0.0100 | 1 | 0.9250 | 0.0750 |
|  | PM | 122 | 0.8525 | 104 | 0.1311 | 16 | 0.0164 | 2 | 0.9180 | 0.0820 |
|  | PW | 135 | 0.9111 | 123 | 0.0889 | 12 | 0.0000 | 0 | 0.9556 | 0.0444 |
|  | SF | 174 | 0.9253 | 161 | 0.0690 | 12 | 0.0057 | 1 | 0.9598 | 0.0402 |
|  | U | 35 | 0.9143 | 32 | 0.0857 | 3 | 0.0000 | 0 | 0.9571 | 0.0429 |
|  | Total | 714 | 0.9104 | 650 | 0.0840 | 60 | 0.0056 | 4 | 0.9524 | 0.0476 |
|  |  |  | CC |  | CT |  | TT |  | C | T |
| SNP 4 | A | 151 | 0.9272 | 140 | 0.0662 | 10 | 0.0066 | 1 | 0.9603 | 0.0397 |
|  | B | 102 | 0.7843 | 80 | 0.1961 | 20 | 0.0196 | 2 | 0.8824 | 0.1176 |
|  | PM | 129 | 0.8217 | 106 | 0.1783 | 23 | 0.0000 | 0 | 0.9109 | 0.0891 |
|  | PW | 136 | 0.5441 | 74 | 0.3529 | 48 | 0.1029 | 14 | 0.7206 | 0.2794 |
|  | SF | 178 | 0.3315 | 59 | 0.6067 | 108 | 0.0618 | 11 | 0.6348 | 0.3652 |
|  | U | 35 | 0.7429 | 26 | 0.2286 | 8 | 0.0286 | 1 | 0.8571 | 0.1429 |
|  | Total | 731 | 0.6635 | 485 | 0.2969 | 217 | 0.0397 | 29 | 0.8119 | 0.1881 |
|  |  |  | CC |  | CG |  | GG |  | C | G |
| SNP 5 | A | 148 | 0.9595 | 142 | 0.0405 | 6 | 0.0000 | 0 | 0.9797 | 0.0203 |
|  | B | 101 | 0.8317 | 84 | 0.1584 | 16 | 0.0099 | 1 | 0.9109 | 0.0891 |
|  | PM | 129 | 0.7829 | 101 | 0.1938 | 25 | 0.0233 | 3 | 0.8798 | 0.1202 |
|  | PW | 136 | 0.8971 | 122 | 0.1029 | 14 | 0.0000 | 0 | 0.9485 | 0.0515 |
|  | SF | 177 | 0.9153 | 162 | 0.0791 | 14 | 0.0056 | 1 | 0.9548 | 0.0452 |
|  | U | 36 | 0.9167 | 33 | 0.0833 | 3 | 0.0000 | 0 | 0.9583 | 0.0417 |
|  | Total | 727 | 0.8858 | 644 | 0.1073 | 78 | 0.0069 | 5 | 0.9395 | 0.0605 |
|  |  |  | GG |  | AG |  | AA |  | G | A |
| SNP 6 | A | 151 | 0.9205 | 139 | 0.0795 | 12 | 0.0000 | 0 | 0.9603 | 0.0397 |
|  | B | 103 | 0.9903 | 102 | 0.0097 | 1 | 0.0000 | 0 | 0.9951 | 0.0049 |
|  | PM | 132 | 0.9545 | 126 | 0.0455 | 6 | 0.0000 | 0 | 0.9773 | 0.0227 |
|  | PW | 138 | 0.6159 | 85 | 0.3768 | 52 | 0.0072 | 1 | 0.8043 | 0.1957 |
|  | SF | 180 | 0.9722 | 175 | 0.0278 | 5 | 0.0000 | 0 | 0.9861 | 0.0139 |
|  | U | 36 | 1.0000 | 36 | 0.0000 | 0 | 0.0000 | 0 | 1.0000 | 0.0000 |
|  | Total | 740 | 0.8959 | 663 | 0.1027 | 76 | 0.0014 | 1 | 0.9473 | 0.0527 |
|  |  |  | CC |  | CT |  | TT |  | C | T |
| SNP 7 | A | 120 | 0.4417 | 53 | 0.4250 | 51 | 0.1333 | 16 | 0.6542 | 0.3458 |
|  | B | 84 | 0.5119 | 43 | 0.3452 | 29 | 0.1429 | 12 | 0.6845 | 0.3155 |
|  | PM | 99 | 0.4747 | 47 | 0.4141 | 41 | 0.1111 | 11 | 0.6818 | 0.3182 |
|  | PW | 124 | 0.2742 | 34 | 0.5000 | 62 | 0.2258 | 28 | 0.5242 | 0.4758 |
|  | SF | 141 | 0.1631 | 23 | 0.5390 | 76 | 0.2979 | 42 | 0.4326 | 0.5674 |
|  | U | 32 | 0.4063 | 13 | 0.4063 | 13 | 0.1875 | 6 | 0.6094 | 0.3906 |
|  | Total | 600 | 0.3550 | 213 | 0.4533 | 272 | 0.1917 | 115 | 0.5817 | 0.4183 |
