# Supplementary material for: Polymorphisms of FST gene and their association with wool quality traits in Chinese Merino sheep
Source: PLoS One. 2017 Apr 6;12(4):e0174868. doi: 10.1371/journal.pone.0174868 (PMC5383234; doi:10.1371/journal.pone.0174868)
Supplement: S3 Table — (DOCX) [file pone.0174868.s003.docx]

**S3 Table. Haplotype frequencies of the identified SNPs in Chinese Merino sheep (Junken Type)**

| Haplotype | Allele combination | Number | Frequency | Haplotype | Allele combination | Number | Frequency |
| --- | --- | --- | --- | --- | --- | --- | --- |
| Haplotype 1 | GG | 1008 | 0.7478 | Haplotype 4 | CC | 1069 | 0.8062 |
|  | GA | 267 | 0.1981 |  | TC | 200 | 0.1508 |
|  | AG | 66 | 0.0490 |  | CG | 57 | 0.0430 |
|  | AA | 7 | 0.0052 | Haplotype 5 | CG | 1227 | 0.8943 |
| Haplotype 2 | GA | 1009 | 0.7621 |  | GG | 73 | 0.0532 |
|  | AA | 259 | 0.1956 |  | CA | 72 | 0.0525 |
|  | GC | 55 | 0.0415 | Haplotype 6 | GC | 614 | 0.5405 |
|  | AC | 1 | 0.0008 |  | GT | 453 | 0.3988 |
| Haplotype 3 | AC | 1073 | 0.8141 |  | AC | 69 | 0.0607 |
|  | AT | 200 | 0.1517 |  |  |  |  |
|  | CC | 45 | 0.0341 |  |  |  |  |
