# Supplementary figures and images for: Polymorphisms of FST gene and their association with wool quality traits in Chinese Merino sheep
Source: PLoS One. 2017 Apr 6;12(4):e0174868. doi: 10.1371/journal.pone.0174868 (PMC5383234; doi:10.1371/journal.pone.0174868)

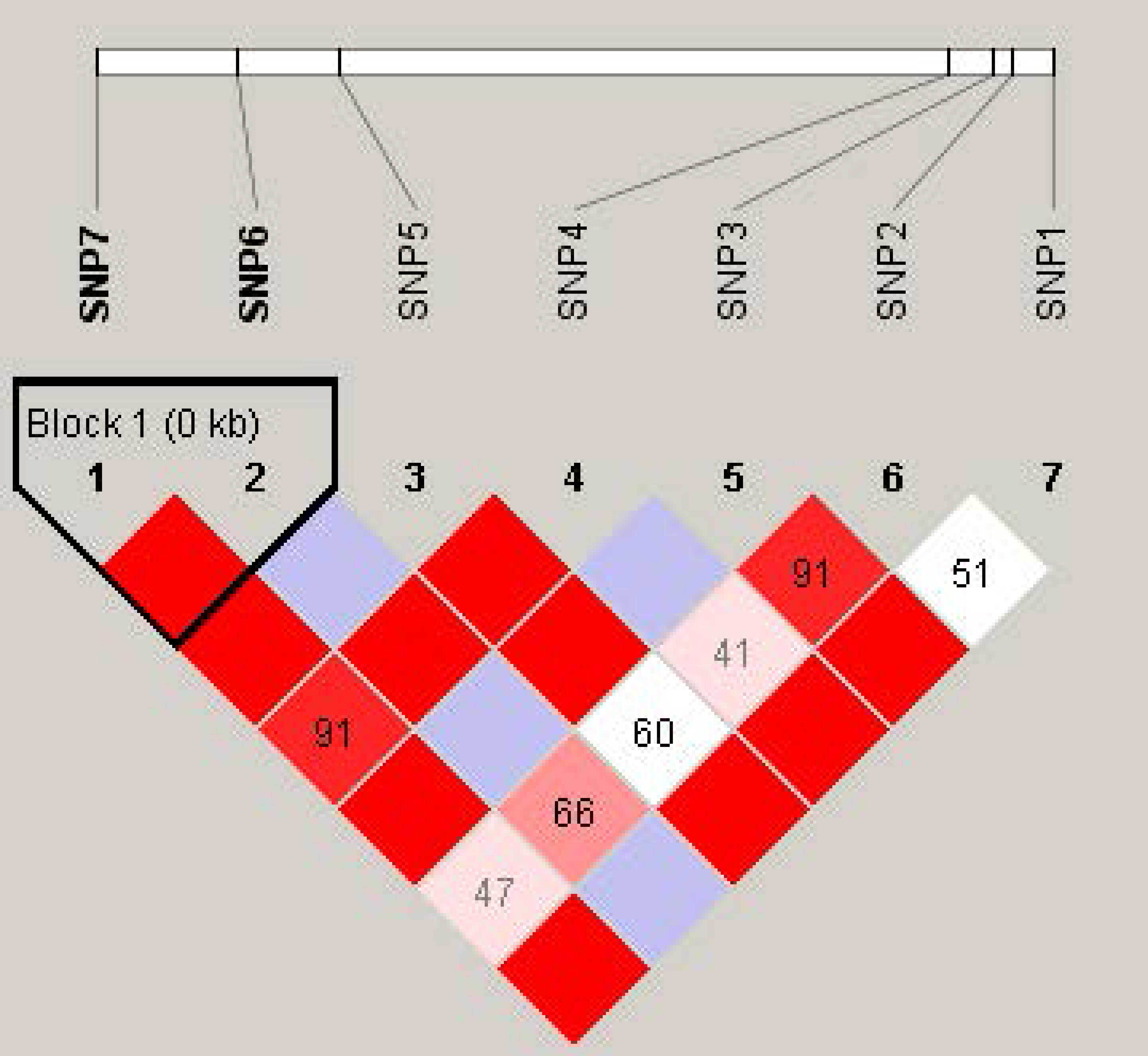

Supplement: S1 Fig — (TIF) [file pone.0174868.s004.tif]
